# Insights into the food safety implications of a commercial *Bacillus thuringiensis* product in hydroponic systems

**DOI:** 10.1128/aem.00885-26

**Published:** 2026-07-10

**Authors:** Michelle Mei Zhen Ten, Nura Arifin-Wong, Lydia Jia Yin Tan, Sanjay Swarup, Dan Li

**Affiliations:** 1 Department of Food Science and Technology, National University of Singapore 37580, Singapore, Singapore; 2 Research Centre on Sustainable Urban Farming, National University of Singapore 37580, Singapore, Singapore; 3 Singapore Centre for Environmental Life Sciences Engineering, National University of Singapore 37580, Singapore, Singapore; 4 NUS Graduate School, National University of Singapore 37580, Singapore, Singapore; 5 Department of Biological Sciences, National University of Singapore 37580, Singapore, Singapore; 6 NUS Environmental Research Institute, National University of Singapore 596762, Singapore, Singapore; University of Georgia Center for Food Safety, Griffin, Georgia, USA

**Keywords:** hydroponics, *B. thuringiensis*, enterotoxin, biocontrol, biofertilizer, contamination, microbiome

## Abstract

This study examined the food safety implications of a commercial *Bacillus thuringiensis* (Bt) product by assessing its pathogenicity and impact on the microbiological quality of crops grown in hydroponic systems. *B. thuringiensis* B3 isolated from the product showed genomic similarity to the foodborne pathogen *Bacillus cereus*, as it possessed complete sets of enterotoxin-encoding genes (*cytK*, *nheABC*, and *hblCDAB*). Caco-2 cytotoxicity assay demonstrated *in vitro* cytotoxicity as B3 cell-free supernatant reduced cell viability at 20% (vol/vol). The food safety implications of Bt product treatment in hydroponic systems cultivating lettuce were monitored for 5 weeks with polymyxin pyruvate egg yolk mannitol bromothymol blue agar to enumerate *B. cereus* group populations. Throughout the cultivation period, *B. cereus* group populations were significantly greater in reservoirs and surfaces of treated systems than controls (*P* < 0.05), which demonstrated sustained elevation of *B. cereus* group populations associated with Bt product application. In contrast, the populations on edible lettuce parts remained at 3.46 ± 0.35 log CFU/g, which was insignificantly different from the control group (*P* > 0.05). Changes in the lettuce leaf microbiome composition and functions also appeared unlikely to compromise food safety. Overall, these results indicate that Bt product usage in hydroponics may increase system-wide *B. cereus* group, but the population levels in the edible parts remain below 5 log CFU/g, the threshold associated with diarrhea syndrome. Moreover, 16S rRNA gene analysis of the Bt product revealed the presence of non-*Bacillus* genera, emphasizing the importance of quality control measures for microbial plant-beneficial products.

**IMPORTANCE:**

Safety evaluations of biologically derived fertilizers and control agents are essential to ensure that crops grown in treated systems are safe for consumption. This includes an assessment of the production composition for contaminants, the potential of the intended organism to cause foodborne illnesses, and any effects on the microbiological quality of the crop. These considerations are especially critical in hydroponic systems, where the recirculating system can amplify the spread and persistence of applied products. In this study, we investigated the safety of a commercial *Bacillus thuringiensis* product due to its widespread use in agriculture and close genomic similarity to the foodborne pathogen *Bacillus cereus*. Our findings underscore the importance of considering the food safety implications when applying biological products in hydroponics and lay the groundwork for safety evaluations of these inputs in food production systems.

## INTRODUCTION

Biofertilizers and biocontrol agent products have been gaining traction as sustainable solutions for improving crop productivity or combating pests and pathogens (1). However, recent concerns have emerged regarding the potential pathogenicity of microorganisms used in these products, as traits associated with rhizosphere colonization can also be involved in human infections (2). Another issue is the contamination of these products due to poor manufacturing practices. A study by Herrmann et al. (3) highlights the high prevalence of contamination of biofertilizer products, revealing that over 50% of the 65 products screened contained at least one species not listed in the product claims. Thus, there have been calls for greater emphasis on safety evaluations of these microorganisms and their associated products (2). This includes implementing more stringent production procedures, conducting strain-level pathogenicity assessments, and adopting workflows such as the Environment and Human Safety Index (1, 4, 5).

*Bacillus thuringiensis* (Bt) is a spore-forming, gram-positive bacterium recognized for its plant growth promotion and biopesticide properties (6, 7). It belongs to the *Bacillus cereus sensu lato* group, which also includes other pathogenic species such as *B. cereus* and *Bacillus anthracis*. Due to its close phylogenetic relationship with the foodborne pathogen *B. cereus*, Bt often harbors genes that encode pore-forming enterotoxins responsible for the diarrhea syndrome caused by *B. cereus*. These toxins include cytotoxin K, non-hemolytic enterotoxin (Nhe), and hemolysin BL (Hbl) (8). Toxico-infections can occur when food containing *B. cereus* surpasses the infectious dose of 10^5^ CFU/g vegetative cells or spores. The spores can survive passage through the gastrointestinal tract and germinate in the intestine, where the germinated vegetative cells produce enterotoxins that lyse epithelial intestinal cells, leading to diarrhea (9). As Bt and *B. cereus* are not differentiated in most diagnostic laboratories, foodborne outbreaks linked to Bt may be underreported. Retrospective evaluations of previously reported *B. cereus* foodborne outbreaks suggest that some of these outbreaks may have been caused by Bt instead (10).

A review by T. De Bock et al. (8) assessed that the populations of Bt on harvested field-grown crops sprayed with Bt are unlikely to exceed the infectious dose. This is because the population tends to decrease post-application due to external factors such as rain wash-off or spore degradation by sunlight exposure. However, these findings may not apply to other application methods. In hydroponic systems, Bt may be applied to the water reservoir instead for its biopesticide properties to reduce mosquito populations, biocontrol properties to manage fungal infections, or as a plant growth-promoting microbe (11–13). In these cases, Bt may wind up on the leaves by translocating from the roots, and these internalized cells could be more resistant to the external factors mentioned (14–16). The persistence of Bt on the leaves could lead to further safety concerns, as a prior study by Wang et al. (17) demonstrated changes to the leaf microbiome following Bt application. It is imperative to characterize these potential hazards, given the importance of hydroponics in urban agriculture due to its greater space efficiency compared to conventional soil agriculture.

To shed light on the potential virulence of Bt and the ambiguity of the safety implications of its usage in soilless systems, we investigated the microbial safety of a commercially available Bt product and its application in a hydroponic system. First, the microbial composition of the product was evaluated for the presence of the claimed Bt species and potential contaminants. Bt isolate B3 was then isolated from the product, and its virulence potential was analyzed through genomic analysis and hemolytic tests. Finally, a 5-week investigation was conducted to determine if Bt product application leads to elevations in the *Bacillus cereus* group population in hydroponic systems and in the lettuce crops grown within it. The impact of the treatment on the microbiome composition and functions of the edible parts was also evaluated through 16S rRNA analysis of the harvested leaves.

## RESULTS

### Microbial composition analysis indicates the presence of unexpected genera within Bt commercial product

**Fig 1:**
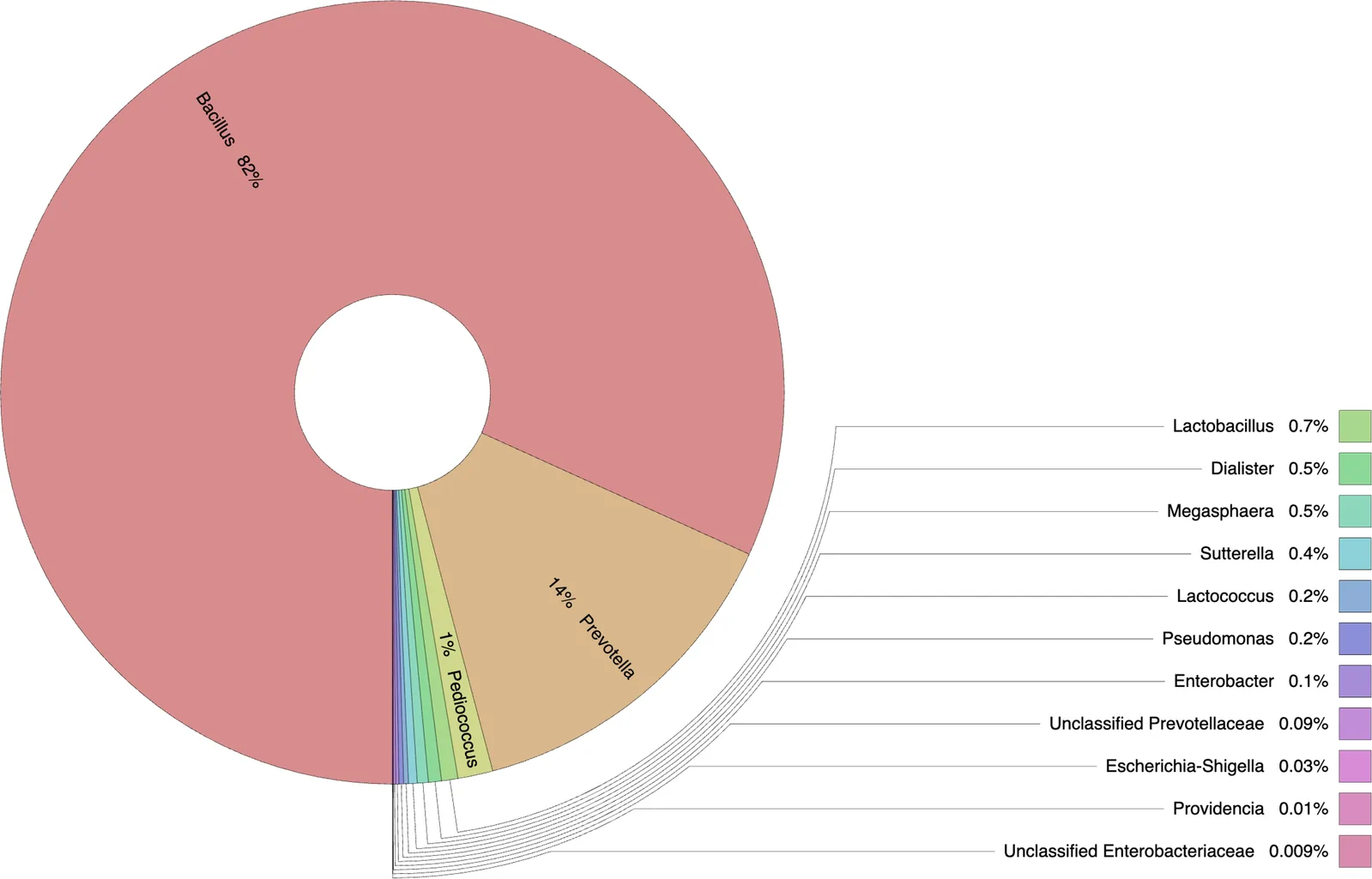
Krona chart illustrating the relative abundance of the microbial community present in the *Bacillus thuringiensis* commercial product at the genus level. Numbers indicate the relative abundance of genera in the product.

The microbial composition of the Bt commercial product, as determined through 16S rRNA gene analysis, is presented in Fig. 1. The analysis revealed that the product was predominantly composed of *Bacillus*, which accounted for 81.8% relative abundance. Thirteen other genera were also present, with *Prevotella* being the second most prominent genus at 14.1%, followed by *Pediococcus* at 1.4%. The remaining genera were detected at levels below 1% of the overall composition. Notably, the genera *Enterobacter* and *Escherichia*-*Shigella*, which have been associated with human pathogens, were also identified (18, 19).

### *B. thuringiensis* B3 isolated from the product possessed enterotoxin genes associated with diarrhea syndrome

**Fig 2:**
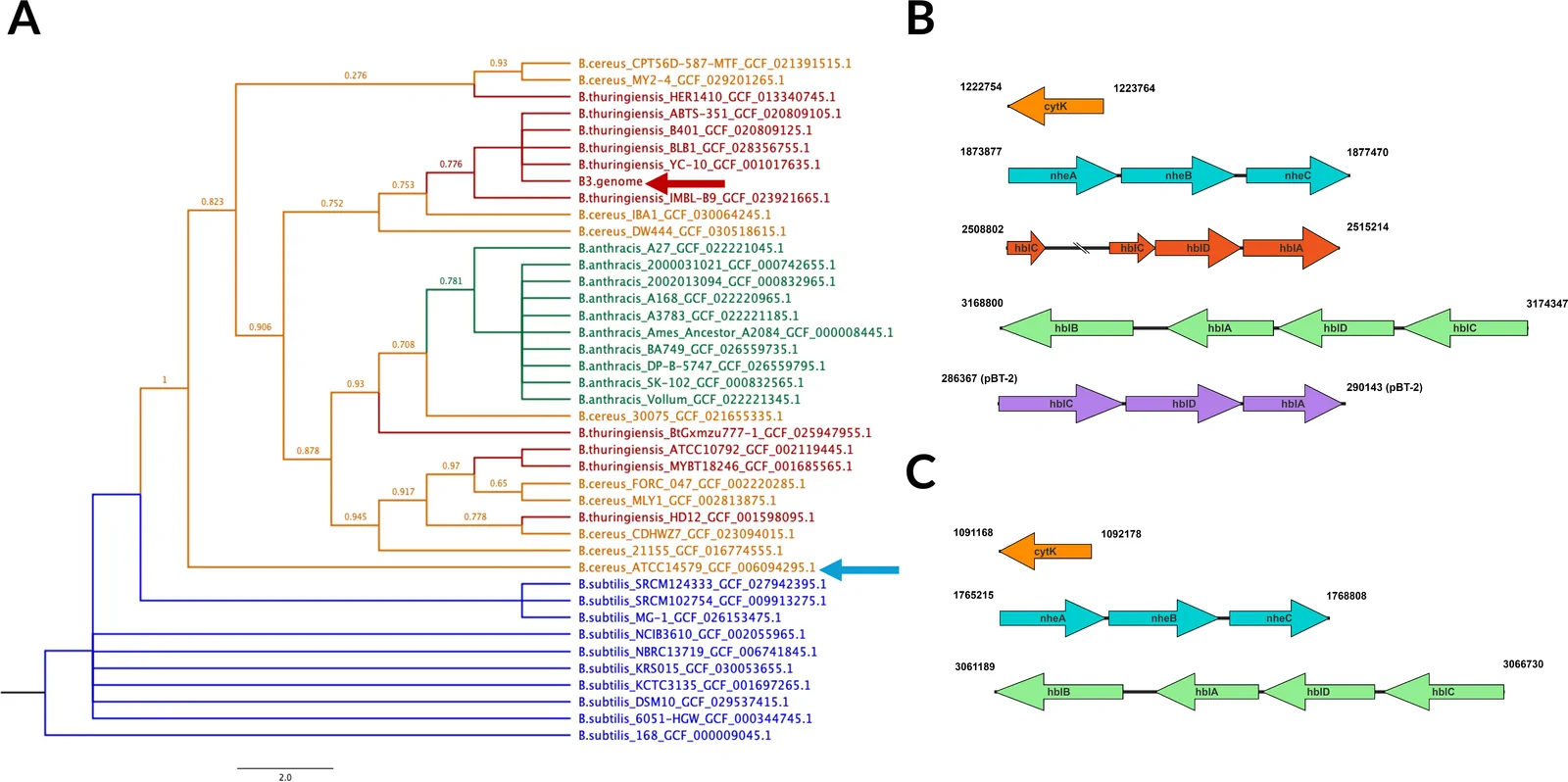
Comparison of genomes between isolate B3 and reference strain *B. cereus* ATCC 14579. (**A**) Phylogenetic tree illustrating the relationship of isolate B3 (red arrow) and reference strain *B. cereus* ATCC 14579 (blue arrow) with closely related members of the *Bacillus* genus. The single-nucleotide polymorphism tree was constructed using the maximum likelihood method on CSI phylogeny. Numbers in the tree represent bootstrap values. (**B**) Gene locus architecture of virulence genes (i.e., *cytK*, *nhe*, and *hbl*) associated with *Bacillus cereus* diarrhea syndrome, as observed in the genomes of B3 isolate and (**C**) the reference strain *B. cereus* ATCC 14579. Numbers indicate the position of genes within the chromosome unless otherwise stated in brackets.

A presumptive Bt (B3) was isolated from the commercial product for further investigation into its microbial safety. The assembled genome of the isolate comprises a circular chromosome and 10 circular plasmids, labeled from pBT-1 to pBT-10. The chromosome had a length of 5,708,468 bp with a GC content of 35.29%, while the plasmid genomic lengths ranged from 5,535 bp to 418,534 bp. Additional characteristics of the genome are detailed in Table S1. A phylogenetic analysis was conducted against publicly available genomes of the *B. cereus* groups, Bt, *B. cereus*, and *B. anthracis*. The resulting phylogenetic tree, shown in Fig. 2A, placed B3 in a clade with other Bt strains. However, the distinction between Bt and *B. cereus* was not as pronounced as that observed for the *B. anthracis* and *Bacillus subtilis* clades. Further investigation into its genome identified seven crystal insecticidal toxin genes (i.e., *cry1Ca15*, *cry1Da1*, *cry2Ab1*, *cry1Aa12*, *cry1Ia10*, *cry9Ea1*, and *cry1Ac1*) within the isolate’s plasmids (Table S2), which indicates its identity as Bt.

The diarrhea syndrome caused by *B. cereus* is attributed to enterotoxins cytotoxin K (encoded by the *cytK* gene), non-hemolytic enterotoxin (Nhe), and hemolysin BL (Hbl). Nhe and Hbl are tripartite proteins, each consisting of three components. The components of Nhe (A, B, and C) are encoded by *nheA*, *nheB*, and *nheC*, respectively, while Hbl components (L2, L1, and B) are encoded by *hblC*, *hblD*, and *hblA*, respectively (9). The *hbl* operon often includes *hblB*, which encodes the Hbl B′ component and is located downstream of the *hblA* gene (20). The gene locus architecture of these genes within the B3 genome is illustrated in Fig. 2B. The B3 chromosome contains one copy of the *cytK* gene (orange) and complete *nhe* (blue) and *hbl* (green) operons. These operons comprised the genes *nheABC* and *hblCDAB*, which were located adjacent to each other within the respective operons. When comparing these three clusters to the gene locus architecture of the *cytK*, *nhe*, and *hbl* genes within the genome of diarrhea reference strain *B. cereus* ATCC 14579, the gene organizations were identical (Fig. 2C). A deeper investigation into the similarity of gene sequences through BLAST comparisons showed low *E* values (0.0), high bitscores (>600), and identity values (>95%) (Table 1). Despite the high similarity, several amino acid additions and non-neutral substitutions were observed in *cytK*, *nheA*, *hblB*, and *hblC* genes.

**TABLE 1:** List of virulence genes annotated within the genome of the *B. thuringiensis* B3 and results of protein BLAST comparisons with corresponding genes in reference strain *B. cereus* ATCC 14579

Operon	Gene	Gene product	Gene location	Start	End	Query cover	*E* value	% identity	Bitscore
NA^*a*^	*cytK*	Cytotoxin K	Chromosome	1222754	1223764	100	0.0	98.81	679
*nhe* operon (blue arrow)	*nheA*	Non-hemolytic enterotoxin A	Chromosome	1873877	1875037	100	0.0	99.48	780
	*nheB*	Non-hemolytic enterotoxin B	Chromosome	1875075	1876283	100	0.0	99.25	801
	*nheC*	Non-hemolytic enterotoxin C	Chromosome	1876391	1877470	100	0.0	99.16	733
*hbl* operon (red arrow)	*hblC*	Hemolysin BL component L2	Chromosome	2508802	2509350	98	3e^−95^	77.09	271
	*hblC*	Hemolysin BL component L2	Chromosome	2512027	2512758	100	1e^−131^	75.72	367
	*hblD*	Hemolysin BL component L1	Chromosome	2512820	2514049	99	0.0	85.71	710
	*hblA*	Hemolysin BL component B	Chromosome	2514081	2515214	100	0.0	68.87	535
*hbl* operon (green arrow)	*hblB*	Hemolysin BL component B’	Chromosome	3168800	3170200	100	0.0	98.50	945
	*hblA*	Hemolysin BL component B	Chromosome	3170576	3171703	100	0.0	99.73	764
	*hblD*	Hemolysin BL component L1	Chromosome	3171740	3172960	100	0.0	99.75	819
	*hblC*	Hemolysin BL component L2	Chromosome	3173022	3174347	100	0.0	96.37	848
*hbl* operon (purple arrow)	*hblC*	Hemolysin BL component L2	Plasmid pBT-2	286367	287686	100	0.0	77.90	673
	*hblD*	Hemolysin BL component L1	Plasmid pBT-2	287749	288978	99	0.0	85.96	711
	*hblA*	Hemolysin BL component B	Plasmid pBT-2	289010	290143	100	0.0	69.23	538

^*a*^ NA indicates not applicable.

Two additional *hbl* operons were annotated within the B3 genome, with one located on the chromosome (red) and the other on the pBT-2 plasmid (purple). These operons comprise the genes *hblCDA*. The *hblC* gene in the red *hbl* operon was unlikely to be functional due to an observed gene insertion. Further BLAST comparisons with the reference *hbl* genes from *B. cereus* ATCC 14579 revealed relatively low identity values (68.87%–85.96%), indicating that the proteins encoded by these clusters have several differences in amino acid sequences compared to the reference strain.

### B3 isolate displayed hemolytic and cytotoxic activity

**Fig 3:**
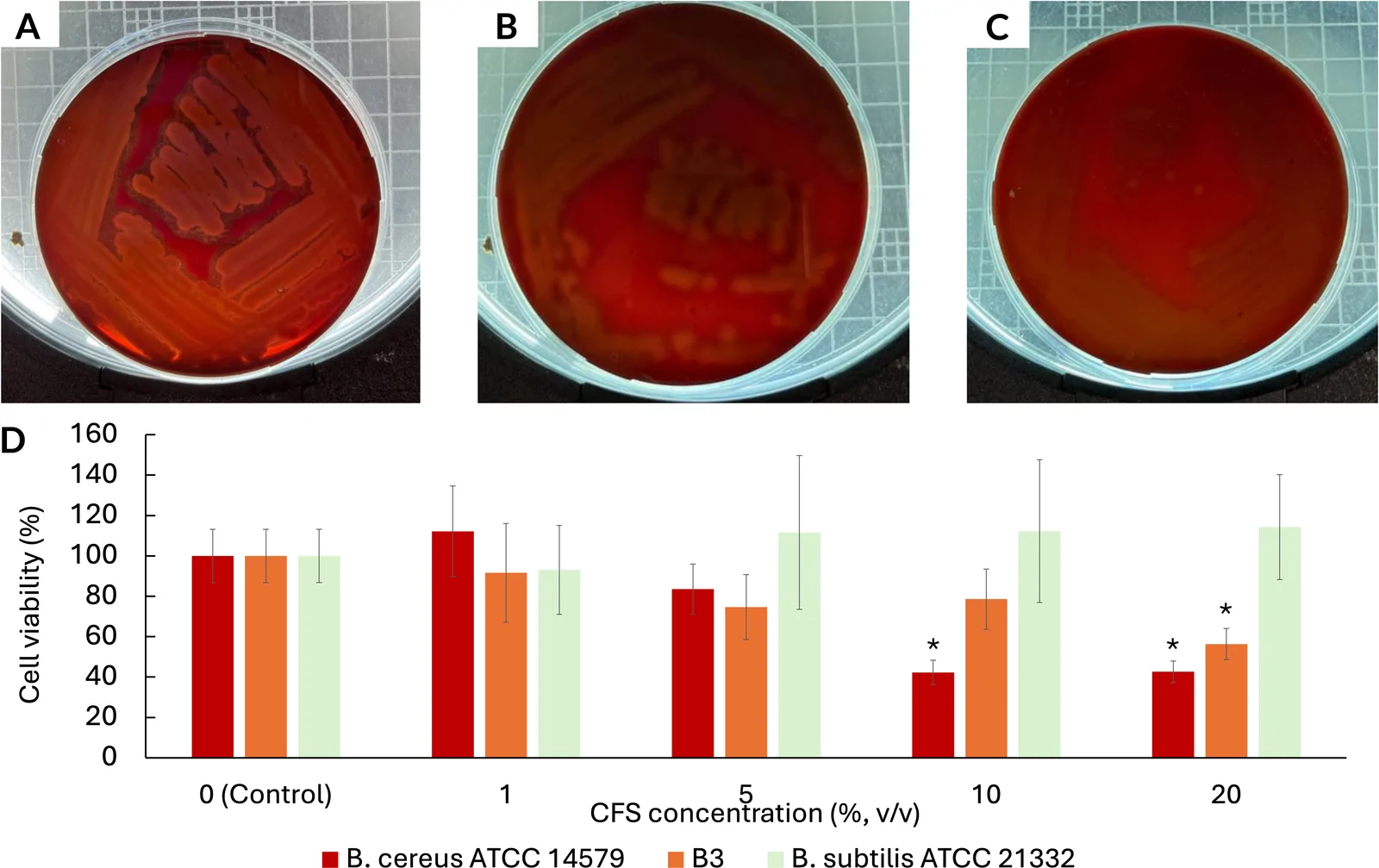
Evaluation of B3 hemolytic ability and cytotoxicity. (**A**) β-Hemolysis by positive control *B. cereus* ATCC 14579. (**B**) α-Hemolysis by B3. (**C**) γ-Hemolysis by negative control *B. subtilis* ATCC 21332. (**D**) Cytotoxicity of cell-free supernatants on Caco-2 cells evaluated with MTT cell proliferation assay. Each datapoint represents the average of three biological replicates with two technical replicates each (*n* = 6). The asterisks (*) indicate significant differences (*P* <  0.05) from control.

The virulence potential of B3 was first evaluated with a hemolytic test on sheep blood agar, with the observations presented in Fig. 3. Hemolysis on blood agar is characterized into three types: β-hemolysis, α-hemolysis, and γ-hemolysis. *B. cereus* ATCC 14579 (positive control) served as a reference for β-hemolysis, as indicated by the clear and complete lysis of cells. In contrast, γ-hemolysis was observed for *B. subtilis* ATCC 21332 (negative control), which was characterized by the absence of clearing of blood agar. B3 showed an intermediate phenotype relative to the two reference strains, with an α-hemolytic reaction marked by incomplete cell lysis as observed by the partial clearing of blood agar.

Among the three *Bacillus* strains, B3 also displayed an intermediate phenotype in the Caco-2 cell cytotoxicity assessment of their cell-free supernatant (CFS). CFSs were tested at varying concentrations, with 0% (vol/vol) treatment serving as the control. Cell viability of the treated cells was then determined through the 3-[4,5-dimethylthiazol-2-yl]-2,5-diphenyltetrazolium bromide (MTT) cell proliferation assay. As seen in Fig. 3D, the differences in toxicity were apparent at 10% and 20% (vol/vol) CFS treatments. At both treatment concentrations, the *B. subtilis* ATCC 21332 CFS showed minimal toxicity, as the cell viability was not significantly different (*P* < 0.05) from control (*B. subtilis:* 119.5% ± 31.7% [10%], 113.1% ± 27.7% [20%]). In contrast, *B. cereus* ATCC 14579 CFS was the most toxic, exhibiting significantly lower (*P* < 0.05) cell viability than control at both concentrations (*B. cereus:* 42.3% ± 6.0% [10%] and 42.6% ± 5.4% [20%]). Meanwhile, the CFS of B3 showed moderate toxicity, as a significantly lower (*P* < 0.05) cell viability was observed only at 20% (vol/vol) treatment (B3: 78.6% ± 14.9% [10%] and 56.3% ± 7.7% [20%]).

Together, the detection of α-hemolysis and a modest reduction in Caco-2 cell viability with B3 at 37°C indicates that it can exhibit hemolytic ability and *in vitro* cytotoxic effects at human physiological temperature, albeit to a lesser extent than the *B. cereus* ATCC 14579 reference strain. The findings motivated further investigation into the food safety implications of Bt product application in a hydroponic system, in which *B. cereus* group populations were enumerated with polymyxin pyruvate egg yolk mannitol bromothymol blue agar (PEMBA).

### Throughout the lettuce cultivation period, the *B. cereus* group population remained elevated in the treated hydroponic system compared to the indigenous population levels

**Fig 4:**
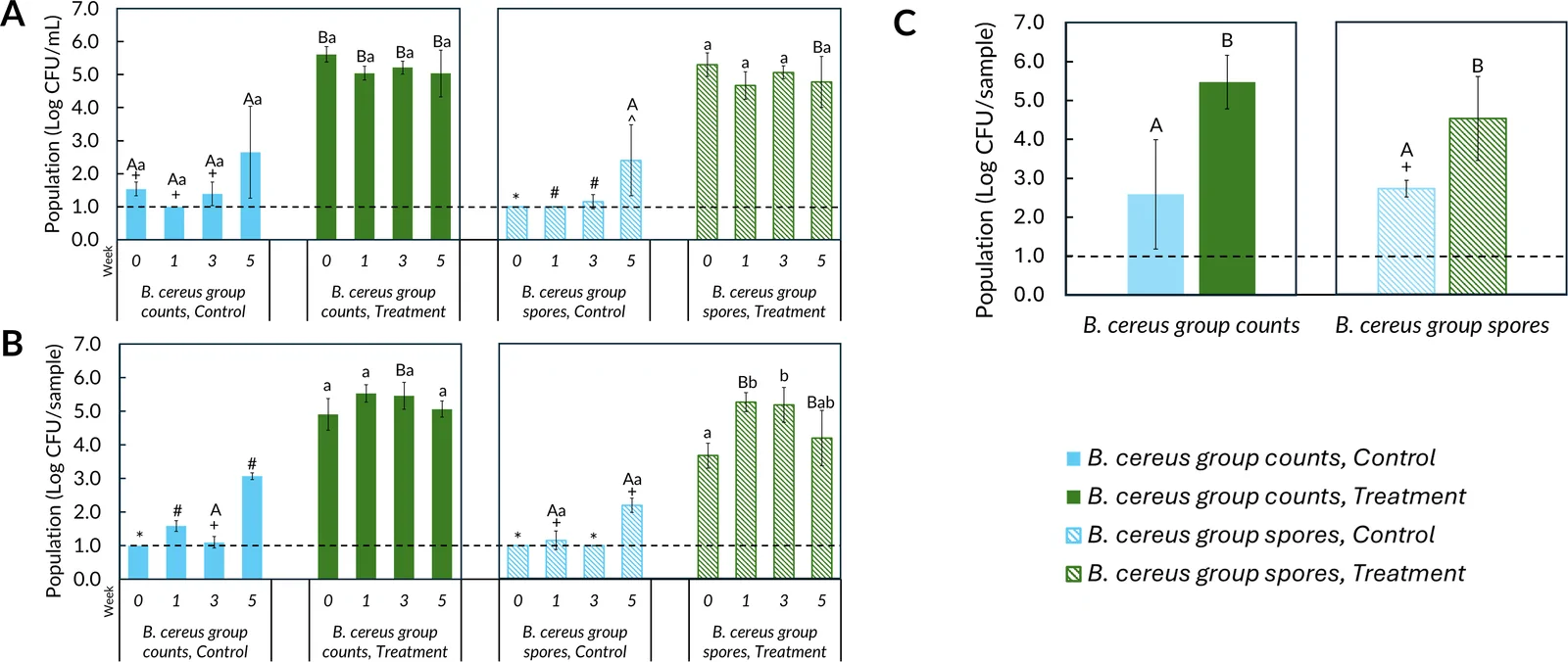
*Bacillus cereus* group population (total counts/spores) in control and treated hydroponic systems, as enumerated on PEMBA. *Bacillus cereus* group population in the (**A**) hydroponic water reservoir and (**B**) wet swab of the system surface (31 cm^2^) over a 5-week operation. (**C**) *Bacillus cereus* group population in the dry swab of the system surface (31 cm^2^) after operation. Each datapoint represents the average of triplicates from two independent experiments, with error bars indicating the standard deviation (*n* = 6 for each group). Lowercase letters (a and b) indicate significant differences (*P* <  0.05) within the same treatment across different weeks, while uppercase letters (A and B) indicate significant differences (*P* <  0.05) between treatments at the same time point. Dotted line represents the limit of detection. Symbols ^, +, #, and * represent 1/6, 3/6, 4/6, and 6/6 replicates falling below the limit of detection, respectively.

Throughout the 5-week operation period, *B. cereus* group levels enumerated on PEMBA plates remained consistent across sampled locations of the hydroponic system in the treatment group. In the hydroponic water reservoir, the *B. cereus* group counts and *B. cereus* group spore populations were at 5.04 ± 0.71 log CFU/mL to 5.62 ± 0.23 log CFU/mL (*P* > 0.05) and 4.67 ± 0.41 log CFU/mL to 5.30 ± 0.36 log CFU/mL (*P* > 0.05), respectively, throughout the 5 weeks (Fig. 4A). On wet surfaces, *B. cereus* group counts were at 4.91 ± 0.48 log CFU/sample to 5.53 ± 0.26 log CFU/sample. While significant changes to the population were detected by repeated measures analysis of variance (ANOVA) (*P* < 0.05), significant pairwise differences were not observed with the Sidak test (adjusted *P* > 0.05), suggesting that the *B. cereus* group counts remained relatively stable on wet surfaces as the changes between time points were not pronounced enough to achieve statistical significance in pairwise comparisons. Meanwhile, the *B. cereus* group spore population on the wet surface increased from 3.68 ± 0.37 log CFU/sample to 5.27 ± 0.28 log CFU/sample (*P* < 0.05) after 1 week of operation before stabilizing at 4.20 ± 0.82 log CFU/sample to 5.19 ± 0.51 log CFU/sample (*P* > 0.05) over the following 4 weeks (Fig. 4B). After 5 weeks of system operation, dry surface swabs showed that the *B. cereus* group counts and spore populations attached to the system surface were 5.48 ± 0.69 log CFU/sample and 4.54 ± 1.08 log CFU/sample, respectively (Fig. 4C).

When the aforementioned *B. cereus* group counts and spore populations in the treated systems were compared to those in the control systems, they were significantly higher (*P* < 0.05) throughout the 5 weeks of system operation. Furthermore, several control samples had population levels below the detection limit (hydroponic water: <1 log CFU/mL, surfaces: <1 log CFU/g). This indicates that Bt product application increased the *B. cereus* group populations in the treated system (hydroponic water and system surfaces) to levels exceeding the indigenous levels, which was sustained throughout the operation period.

### *B. cereus* group population on treated lettuce crops was not higher than indigenous levels

**Fig 5:**
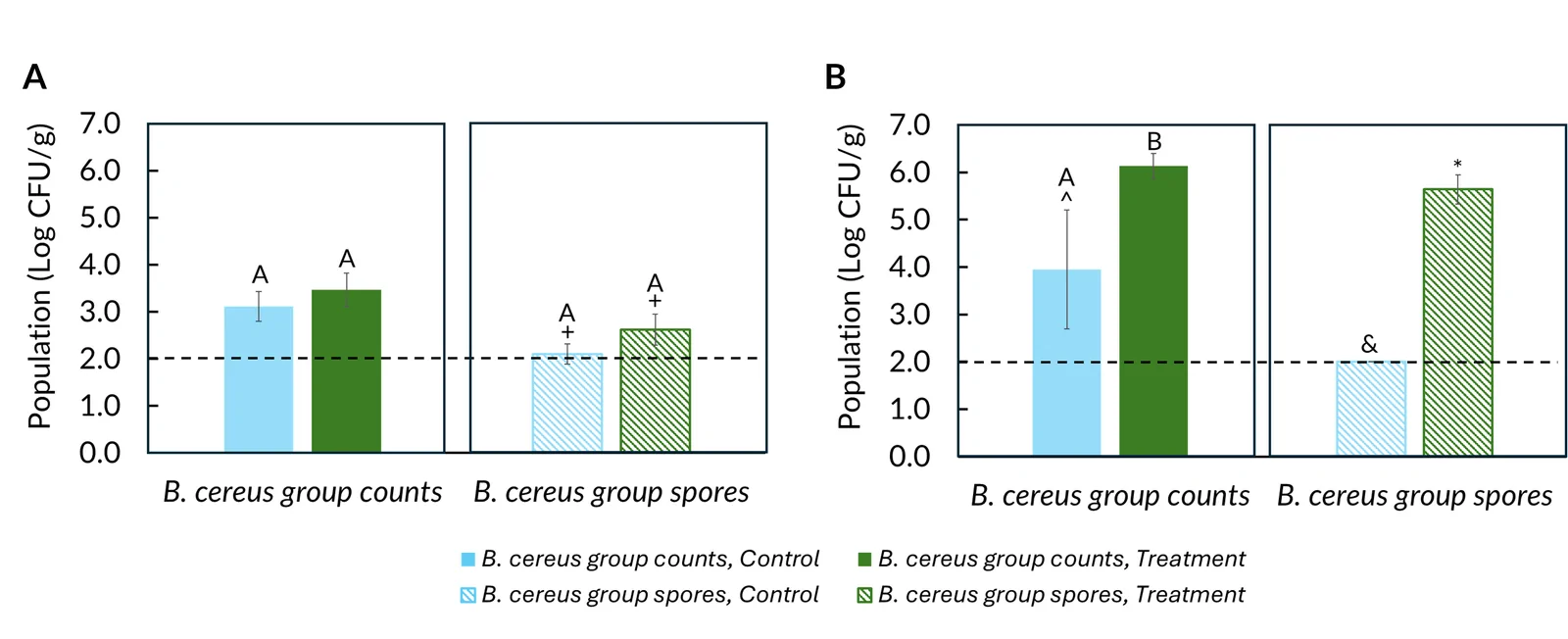
*Bacillus cereus* group population (total counts/spores) in the (**A**) leaves and (**B**) roots of lettuce crops harvested from control and treated hydroponic systems, as enumerated on PEMBA. Each datapoint represents the average of triplicates from two independent experiments, with error bars indicating the standard deviation (*n* = 6 for each group). Different letters indicate significant differences (*P* <  0.05) between treatments. The dotted line represents the limit of detection. Symbols ^, +, and & represent 1/6, 3/6, and 5/6 replicates falling below the limit of detection, respectively. The symbol * represents triplicates from the second independent trial to be below 4 log CFU/g.

During harvest, it was observed that the *B. cereus* group counts and spore populations in the lettuce leaves harvested from the treated system (3.46 ± 0.35 log CFU/g and 2.62 ± 0.33 log CFU/g, respectively) were not significantly different from those harvested from the control systems (3.11 ± 0.31 log CFU/g and 2.10 ± 0.21 log CFU/g, respectively; *P* > 0.05) (Fig. 5A). However, the *B. cereus* group counts on the roots of lettuce grown in the treated system (6.13 ± 0.26 log CFU/g) were significantly higher than those from the control group (3.95 ± 1.26 log CFU/g, *P* <  0.05) (Fig. 5B). Meanwhile, the *B. cereus* group spore population on the roots of treatment plants showed considerable variations between experiments, with 5.64 ± 0.31 log CFU/g enumerated from the first independent trial but below 4 log CFU/g on the subsequent trial for all plants. Overall, these findings indicate that while Bt product application increased the *B. cereus* group population on lettuce roots, it did not lead to a similar increase in the leaves.

### Bt product application altered lettuce leaf microbial composition and function

**Fig 6:**
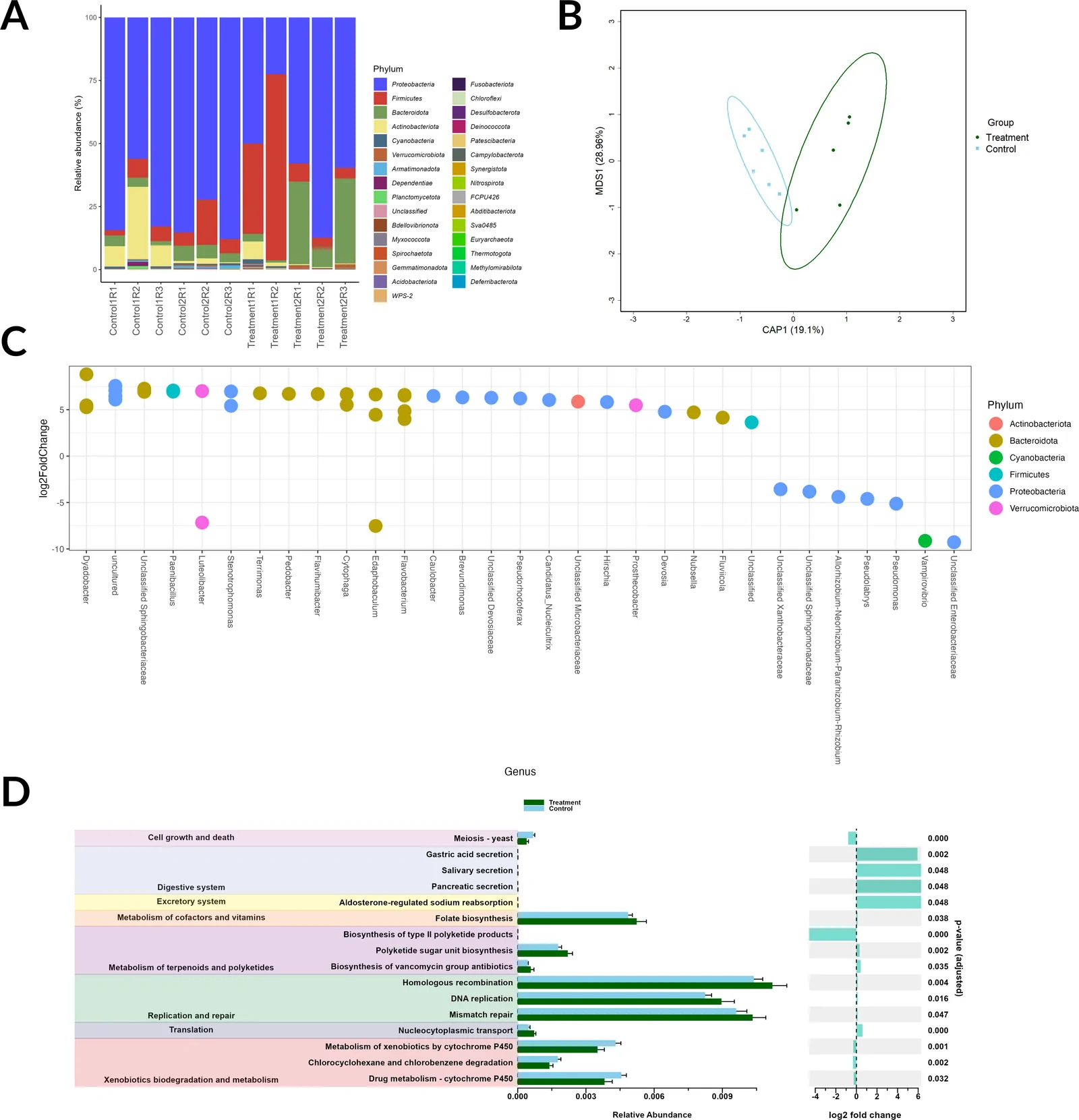
Microbial community analysis of lettuce leaves harvested from control and treatment hydroponic systems in two independent experiments (*n* = 6 for each group), with one treatment sample omitted due to high levels of organelle contamination. (**A**) Taxonomic barplot illustrating the relative abundance of the microbial community at the phylum level. (**B**) Canonical analysis of principal coordinate (CAP) plot showing compositional differences (β-diversity) based on Bray-Curtis distances. Each point represents a microbiome sample from a single lettuce plant, and samples are enveloped based on treatment group. (**C**) Differential abundance analysis of leaf microbial communities between control and treatment groups at the ASV level. ASVs are classified to genera (*x* axis) and phylum level (colors). Positive log2 fold change values indicate a greater abundance in treatment samples, while negative values indicate a greater abundance in control samples. (**D**) Relative abundance and log2 fold change of predicted KEGG functions grouped according to level 2 and 3 categories, which were significantly different between the microbial community of control and treatment (adjusted *P* < 0.05).

A microbial composition analysis was conducted on the harvested lettuce leaves to identify microbiome differences resulting from Bt product treatment. One treatment sample was omitted due to high levels of organelle contamination. The microbial composition of the remaining control and treatment samples at the phylum and genus levels is shown in Fig. 6A and Fig. S1, respectively. In both control and treatment groups, the lettuce leaf microbiome was generally dominated by *Proteobacteria*, *Firmicutes*, *Bacteroidota*, and *Actinobacteriota*. α-Diversity analysis showed no significant differences (*P* >  0.05), possibly due to large variability in species richness, as measured by the Chao1 and Shannon indexes, among the treatment samples (Fig. S2). However, significant composition differences between treated and control groups were identified through permutational multivariate analysis of variance (PERMANOVA) analysis (*R*^2^ = 0.191, *P* = 0.020), as evidenced by the clear separation of control and treatment datapoints in the canonical analysis of principal coordinates (Fig. 6B). This finding was supported by the non-significant result from the permutational multivariate analysis of dispersion (PERMDISP) analysis (*P* = 0.113), which suggests that the differences were not due to variations in dispersion and likely represent genuine differences between the control and treatment groups.

These differences were investigated by performing a differential abundance analysis (DAA) at the amplicon sequencing variant (ASV) level. The analysis identified 33 ASVs that increased and 9 ASVs that decreased as a result of the Bt product treatment (adjusted *P* < 0.05). Most of these ASVs belong to the *Bacteroidota* and *Proteobacteria* phyla (Fig. 6C). Notably, the genera of ASVs observed in the commercial product were not observed to have increased in the leaf microbiome of treated lettuce. Although no ASVs associated with foodborne pathogens were identified in the DAA analysis, several ASVs, all belonging to *Proteobacteria*, were classified under taxa linked to opportunistic pathogens (21–23). Specifically, the relative abundance of *Brevundimonas* and *Stenotrophomonas* was increased, while that of *Pseudomonas* was reduced.

To assess if changes in these ASVs could have altered disease-associated community functions, further analysis was conducted using PICRUSt2. The analysis revealed significant changes to eight predicted KEGG level 2 functions (adjusted *P* < 0.05), none of which were associated with human disease and infections. This suggests that changes in the previously mentioned ASVs may not have direct impacts on human illnesses (Fig. 6D). However, there may be indirect implications for the gut microbiome, as several functional changes to the metabolic-related functions of the microbial communities were observed. Notable changes include an increase in functions related to the metabolism of cofactors and vitamins, particularly in folate biosynthesis, and a decrease in functions associated with xenobiotic biodegradation and metabolism. Meanwhile, for the metabolism of terpenoids and polyketides, there was an increase in the biosynthesis of vancomycin group antibiotics and polyketide sugars, but a decrease in the biosynthesis of type II polyketide products.

## DISCUSSION

In this study, we conducted a comprehensive safety assessment of the Bt plant-beneficial product by examining four key aspects: the level of product contamination, the *in vitro* cytotoxic and hemolytic activity of *B. thuringiensis* B3 isolated from the product, the impact of product application on *B. cereus* group populations in the hydroponic system and crops, and potential changes to the leaf microbiome community and its predicted functions.

The potential of Bt to cause foodborne illnesses has garnered increased attention due to studies reporting its ability to express enterotoxins associated with diarrhea syndrome. Furthermore, retrospective studies have linked previous foodborne outbreaks that were initially attributed to *B. cereus* to Bt instead (10, 24). In this study, *B. thuringiensis* B3 isolated from a commercial product possessed a full genomic set of the pore-forming enterotoxins cytotoxin K, non-hemolytic enterotoxin, and hemolysin BL and demonstrated the ability to damage erythrocytes and reduce the viability of Caco-2 cells. However, these phenotypes were attenuated when compared with *B. cereus* ATCC 14579 despite possessing two additional hemolysin BL gene clusters. In prior studies, the cytotoxicity of Bt was varied across strains, and the degree of cytotoxicity has been linked to differences in the production levels of enterotoxins like Nhe and Hbl (24–26). Hemolytic activity is likewise variable and is linked to not only Nhe and Hbl but also other pore-forming hemolysins, such as hemolysin II (27). The less severe phenotypes observed in B3 may also be due to a reduced functionality of the enterotoxins, as non-neutral differences in amino acid sequences were observed in several genes for each enterotoxin. The impact of these amino acid sequence changes on the functionality of *B. cereus* enterotoxins has yet to be clearly understood (28).

As Bt has demonstrated *in vitro* cytotoxicity in this study and literature, safety evaluations of agricultural applications remain important to characterize potential consumer exposure (8, 24, 26). While the safety of Bt field spray applications has been thoroughly evaluated by De Bock et al. (8), similar safety evaluations in hydroponic applications are limited. This research gap warrants attention as Bt is being explored for biopesticidal, antifungal, and plant growth-promoting beneficial effects, where it is often applied directly to the reservoir (11–13, 29). Our study demonstrated that this practice can lead to the sustained elevation of *B. cereus* group populations throughout the hydroponic system (i.e., water reservoir and system surfaces) until crop harvest. We hypothesize that these elevated counts may result from the persistence of product-associated Bt in the hydroponic system or cooperative mechanisms between the introduced Bt and the indigenous *B. cereus* group population. Such interactions are plausible, as Bt and *B. cereus* do co-exist in environments and can engage in mutualistic processes, such as horizontal gene transfer (30, 31). Future assessments should consider incorporating strain-level monitoring to clarify the mechanisms underlying the observed elevations in *B. cereus* group populations following Bt product application in hydroponic reservoirs.

Bt product treatment was found to increase *B. cereus* group populations attached to system surfaces, which persisted at populations of ~3 to 4 log CFU/cm^2^. Furthermore, an increase in spore counts on wet surface swabs was observed after a week of system operation. *B. cereus* group spore persistence may exacerbate surface attachment behavior as they have hydrophobic surfaces, which readily adhere to hydrophobic materials commonly used in these systems (e.g., polyvinyl chloride) (32). Once attached, the *B. cereus* group can become incorporated in the biofilm growing on these surfaces. Given the evidence suggesting potential cytotoxicity of Bt as well as known pathogenicity of other *B. cereus* group members, it is imperative to ensure the thorough removal of these biofilms between harvest cycles to mitigate its accumulation on the system surfaces. Although biofilms in hydroponic systems are typically removed through cleaning disinfectants, spores within biofilms tend to exhibit high tolerance toward cleaning disinfectants and necessitate more thorough cleaning procedures (32). Overall, this highlights the importance of thoroughly sanitizing system surfaces in Bt applied hydroponic systems post-harvest to mitigate associated risks.

Despite sustained elevation of *B. cereus* group populations in the system and lettuce roots, both traditional culturing and 16S rRNA gene sequencing suggested limited translocation to the leaves. *B. cereus* group populations in the leaves harvested from treated systems remained at about 3 log CFU/g, which was not significantly different from the control group. These observations corroborate with prior studies on Bt spray applications, which reported similar Bt levels on the leaves of different crops, including rice and cabbage (17, 33). Similar leaf population levels were also observed with *B. cereus* that was applied through roots in cabbage (34). Overall, our results suggest that Bt product application to a hydroponic water reservoir is unlikely to increase *B. cereus* group populations on edible leaves rising beyond 5 log CFU/g, the threshold associated with most foodborne outbreaks caused by the *B. cereus* group, as reviewed by EFSA (35).

Although Bt product application did not result in substantial changes in the *B. cereus* group populations in the leaves, our results suggest that it can still influence the leaf microbiome. However, analysis of differentially abundant ASVs and community functions indicates that changes induced by Bt product application did not alter microbiome functions associated with infectious diseases in humans. Our study found that Bt product application increased the relative abundance of *Brevundimonas* and *Stenotrophomonas* while decreasing that of *Pseudomonas*. This variable effect could be due to members of the *B. cereus* group, such as Bt and *B. cereus*, producing antimicrobial secondary metabolites (e.g., bacteriocins), which can modulate microbial communities (36–39). While studies have demonstrated that secondary metabolites from Bt and *B. cereus* have antimicrobial effects on the opportunistic pathogen *Pseudomonas aeruginosa*, their impact on *Brevundimonas* and *Stenotrophomonas* is not well documented (40, 41). Our findings highlight a knowledge gap regarding how the *B. cereus* group affects various genera, such as the selectivity of their secondary metabolites. Elucidating this is important for assessing the overall impact of the Bt product on microbiome composition of hydroponic systems and food microbiological safety of the crops produced. Understanding these effects would also aid in explaining the community changes observed in our study that led to alterations in metabolic functions. Such changes could influence the gut microflora if the microbial community members survive passage through the gastrointestinal tract after lettuce consumption. Currently, there is a research gap regarding the impacts of the vegetable leaf microbiome on the gut microbiome, which future studies could explore (42).

Apart from evaluating the safety of plant-beneficial microorganisms sold as biofertilizer and biocontrol products, it is crucial to ensure these products are free from contaminants, especially since they are used for food production. In the evaluated product, we identified not only *Bacillus* but also 13 other genera, including potential pathogens such as *Escherichia*-*Shigella*. Foodborne pathogens under this genus, such as pathogenic *Escherichia coli* and *Shigella*, have previously caused foodborne outbreaks related to fresh produce (43, 44). These findings are consistent with prior studies that have found several commercial biofertilizers to be contaminated, some with potentially pathogenic genera, including *Escherichia-Shigella*, *Staphylococcus*, and *Clostridium* (3, 45). The presence of contaminants is concerning because pathogens and opportunistic pathogens can be unintentionally introduced to crops for consumption. Although the leaf microbiome analysis in the current study did not show evidence of contaminants colonizing the leaves, it is still crucial to minimize the exposure to potential pathogens in food production. As these unexpected bacteria were likely introduced during product manufacturing or storage, biofertilizer and biocontrol production facilities should adhere to good manufacturing practices to minimize contamination risks from workers (1).

In conclusion, our study suggests that applying a Bt plant-beneficial product to hydroponic reservoirs may lead to a sustained, system-wide elevation of *B. cereus* group populations. However, the lettuce grown in these systems contained *B. cereus* group populations below the thresholds that may pose a food safety concern. Nevertheless, measures are recommended to minimize cross-contamination between the edible parts of crops and other hydroponic components with high *Bacillus cereus* group loads, such as the hydroponic system inner surfaces, water reservoir, and crop roots. Furthermore, treated hydroponic systems should be accompanied by stringent cleaning protocols that effectively remove spores from the system. Future research can help clarify how variation in Bt strains’ cytotoxic phenotypes relates to their ability to cause diarrhea syndrome and the associated minimum infectious dose. Once sufficient data are available, a quantitative risk assessment incorporating strain-level monitoring should be conducted. The presence of genera other than *Bacillus* in the plant-beneficial product suggests food safety concerns from another perspective, underscoring the need for quality control of biofertilizer and biocontrol products. Overall, this study highlights key safety considerations for microbial plant-beneficial product usage in hydroponic systems and lays the groundwork for future safety evaluations of these products in food production systems.

## MATERIALS AND METHODS

### Investigation of microbial composition of Bt commercial product by 16S rRNA gene analysis

A commercially available Bt product was purchased from MarkNature (https://marknature.com/products/bacillus-thuringiensis?srsltid=AfmBOooyL95nv1-cAzWjE1wENj6JE8FFYDol9BhqPQiVr4nU7B_rmh6e), and its microbial composition was investigated through 16S rRNA gene analysis. The product (0.1 g) was resuspended and washed in phosphate-buffered saline (PBS) solution (Vivantis Technologies, Petaling Jaya, Malaysia) before being spun down at 9,000 × *g* for 5 min at 4°C (Centrifuge 5810 R; Eppendorf AG, Germany) to obtain a pellet. DNA extraction from the pellet was carried out with the GeneJET Genomic DNA Purification Kit (Thermo Fisher Scientific, Vilnius, Lithuania) according to the manufacturer's guidelines. The quality and quantity of the extracted DNA were assessed with BioDrop DUO (BioDrop, Cambridge, UK) and sent to Azenta US, Inc. for sequencing. The V3 and V4 regions of the 16S rRNA gene were amplified with the forward primer (5′ CCTACGGRRBGCASCAGKVRVGAAT 3′) and the reverse primer (5′ GGACTACNVGGGTWTCTAATCC 3′). Paired-end sequencing with 250 bp reads was performed on the Illumina NovaSeq platform according to the manufacturer’s instructions.

Raw sequencing data were processed on the open-source Quantitative Insights into Microbial Ecology (version 2024.2) (QIIME 2) pipeline (46). Forward and reverse primers and adaptors were trimmed, and low-quality bases were removed with cutadapt (quality threshold  <30 Phred score) (47). The high-quality reads were then merged using VSEARCH (48). Sequences that lacked primers or failed to merge were removed in these steps. Next, the reads underwent processing with deblur (49) to denoise sequences and remove chimeras. The resulting ASVs of 250 bp were classified using a taxonomic classifier trained using the Silva database (version 138) (50). Finally, the classified ASVs were used to construct a Krona chart at the genus level with the Krona chart plugin (51).

### Isolation and identification of presumptive *B. thuringiensis* B3 from a commercial product

Commercial product (0.1 g) was resuspended in 10 mL of PBS and streaked onto PEMBA (Oxoid, Hampshire, UK). This agar is designed to selectively isolate *B. cereus* and other closely related species, including *B. thuringiensis*. The agar plates were incubated at 30°C for 24 h. Blue colonies that grew on the agar indicate a lack of mannitol fermentation and are presumptive Bt colonies. A singular blue colony was selected and cultured for 24 h in Luria-Bertani (LB) broth (Oxoid) at 30°C to obtain a working culture. The isolate was then streaked on LB agar (Oxoid) for storage at 4°C.

DNA was extracted from an overnight culture of the bacteria isolate as described above and sent to Azenta US, Inc., for third-generation whole-genome sequencing and assembly. Briefly, short- and long-read sequencing was performed on Illumina PE150 platform and PacBio Sequel system, respectively. The PacBio HiFi reads obtained were assembled using Hifiasm (version 0.13-r308) (52) and Canu (version 1.7) (53), followed by polishing with Pilon (version 1.22) (54).

Preliminary prediction of the isolate species was conducted using Kmer algorithm-based software KmerFinder at the Center of Genomic Epidemiology (CGE) (55–57). To determine the phylogeny of the isolate relative to known species of the *B. cereus* group (i.e., *B. anthracis*, *B. cereus*, and Bt), a single-nucleotide polymorphism-based CSI phylogeny tool at CGE (58) was used to construct a phylogenetic tree of the previously assembled isolate genome and publicly available genomes of *B. anthracis*, *B. cereus*, and Bt obtained from the National Center for Biotechnology Information (NCBI) database. Publicly available genomes of *B. subtilis* were used as an outgroup. The resulting phylogenetic tree was visualized on FigTree 1.4.4. All bioinformatic analyses were performed using default parameters of the software. Prokka (version 1.14.6) (59) and CryProcessor (60) were used for the identification of cry genes within the assembled genome.

### Analysis of virulent genes of B3 isolate

The assembled B3 isolate genome was annotated by the Bacterial and Viral Bioinformatics Resource Center (https://www.bv-brc.org). Genes annotated as *hbl*, *nhe*, or *cytK* were compared to those present in the reference strain *Bacillus cereus* Frankland and Frankland ATCC 14579 (accession no. GCF_006094295.1) using NCBI protein BLAST with default parameters. The gene locus architecture of *hbl*, *nhe*, and *cytK* gene sequences in both B3 isolate and *B. cereus* ATCC 14579 was illustrated using Illustrator for Biological Sequences 2.0 (61).

### Analysis of the hemolytic ability of B3 isolate

For the hemolysis test, *B. cereus* ATCC 14579 and *B. subtilis* (Ehrenberg) Cohn ATCC 21332 were used as the positive and negative controls, respectively. Both strains were purchased from ATCC and were activated in LB broth (Oxoid) for two passages at 37°C for 24 h at 120 rpm. The B3 isolate was cultured under the same conditions but at 30°C instead. The overnight culture of each strain was streaked onto blood agar base supplemented with 7% defibrinated sheep blood (Oxoid) and incubated at 37°C for 24 h.

### Analysis of cytotoxicity on Caco-2 cells with MTT assay

The cytotoxicity of the cell-free supernatant of B3, *B. subtilis* ATCC 21332, and *B. cereus* ATCC 14579 on Caco-2 cells was evaluated. First, the three *Bacillus* strains were inoculated in brain-heart infusion broth with 0.1% (wt/wt) glucose (BHI-G, Oxoid) at an optical density of 0.2 measured at 595 nm and cultured at 37°C for 6 h at 150 rpm. Subsequently, these cultures were centrifuged at 9,000 × *g* at 4°C for 5 min. The resulting supernatants were then filter sterilized with 0.22 µm membrane filters and kept frozen for downstream cell toxicity experiments.

Caco-2 cells were purchased from American Type Culture Collection and were cultured in complete Dulbecco’s Modified Eagle Medium (cDMEM; Cytiva, Utah, USA), which comprises cDMEM supplemented with 20% fetal bovine serum (Cytiva, Pasching, Austria) and 1% penicillin-streptomycin solution (Life Technologies, Carlsbad, CA, USA) at 37°C with 5% CO_2_ for 72–96 h until 70%–80% confluence was achieved. Cell density in the T25 flasks was determined from the extrapolation of the average of cell counts made from four 10 μm × 10 µm areas observed under the microscope. The cells were seeded in 96-well microplates containing cDMEM at a density of 2 × 10^4^ cells/well and incubated at 37°C with 5% CO_2_ for 72 h for cell attachment. After incubation, the medium was carefully removed by pipetting and replaced with 200 µL cEMEM containing either B3, *B. subtilis* ATCC 21332, or *B. cereus* ATCC 14579 cell-free supernatants at 1%, 5%, 10%, and 20% (vol/vol). Each treatment was done with three biological replicates. Control (0% vol/vol) wells were treated with cEMEM and 20% BHI-G, while blank wells contained the medium without cells. The plates were once again incubated at 37°C with 5% CO_2_ for 24 h. After incubation, the cells were analyzed with the Cell Proliferation Kit I (Roche, Mannheim, Germany). First, 20 μL MTT labeling reagent was added to each well. The plates were incubated at 37°C with 5% CO_2_ for 4 h, and 100 μL solubilization buffer was then added to the wells for the complete solubilization of formazan crystals. After incubation at 37°C with 5% CO_2_ for 24 h, the absorbance at 570 nm was measured with a microplate reader and corrected with absorbance at 690 nm and baseline absorbance of blanks (Epoch 2; Biotek, Vermont, USA). For each treatment tested, the cell viability was calculated with the equation below:


$$\%\text{ Cell viability}=\frac{\text{Mean corrected sample absorbance at 570 nm}}{\text{Mean corrected control absorbance at 570 nm}}\times 100\%.$$

### Setup of hydroponic system

Grand Rapid lettuce seeds were purchased from an online supplier (Ban Lee Huat Seed Pte Ltd, Singapore). The seeds were first disinfected in 1.2% hypochlorite solution for 12 min, followed by three rinses with sterile deionized water. Germination took place on wet filter paper at 25°C in darkness for 4 days, followed by 3 days under a 12 h light/12 h dark cycle. After 7 days, 12 seedlings were transplanted to a 10 L nutrient film technique hydroponic system. The system used autoclaved lightweight expanded clay aggregates as the substrate and commercially available hydroponic nutrient solution (Power-Gro, SingCrop Services Pte Ltd, Singapore). Electrical conductivity and pH value of the water reservoir were maintained between 1.5 and 1.8 mS/cm and 5.5 and 6.5, respectively. Light was provided using four 10 W light-emitting diode plant-growing lamps, maintaining a 12 h light/12 h dark cycle. The system surface that was in contact with the water reservoir was marked with 15 rectangles, each 31 cm^2^ (6.2 cm × 5 cm), for sampling of the surface *B. cereus* group population. This setup was duplicated to obtain one control (untreated) and one treatment setup inoculated with Bt product at 6 log CFU/mL in the hydroponic water. Between two independent experiments, the system was disinfected and scrubbed with 10% bleach (Clorox, Malaysia), before rinsing twice with tap water.

### Impact on *B. cereus* group population levels in the hydroponic system following Bt product treatment

**Fig 7:**
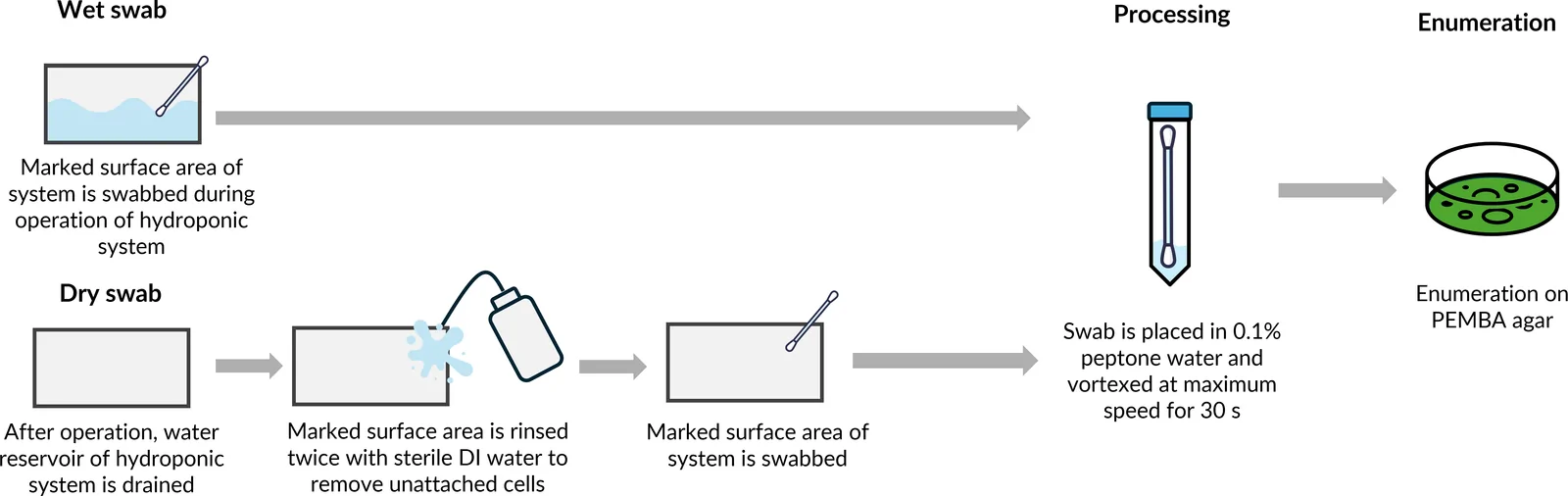
Workflow of sampling *Bacillus cereus* group populations on marked surfaces of the hydroponic system, both during operation (wet swab) and after operation (dry swab).

The hydroponic system was operated for 5 weeks until the harvest of lettuce plants. On weeks 0 (day 0), 1 (day 7), 3 (day 21), and 5 (day 35), triplicate samples of 100 mL hydroponic water from the reservoir and wet swabs of system surfaces were taken from the running hydroponic system. Wet surface swabs were obtained using a sterile cotton swab to sample previously marked surfaces of 31 cm^2^. Each swab was then placed in 1 mL of 0.1% peptone water (Oxoid) before being vortexed at maximum speed for 30 s (Fig. 7). The water and wet-surface swab samples were subsequently plated on PEMBA (Oxoid) and incubated at 30°C for 24 h. Blue colonies observed after incubation indicate a lack of mannitol fermentation and are characteristic of *B. cereus* and *B. thuringiensis* (62). These colonies are denoted as *B. cereus* group counts, which comprise both vegetative and spore cells. For the enumeration of *B. cereus* group spore cells, samples were heated at 80°C for 10 min prior to plating on PEMBA. After 5 weeks, operation of the hydroponic system was terminated for lettuce harvesting and collection of dry surface swab samples. The water reservoir was drained, and dry swabs of the system surfaces were sampled in triplicate. The previously marked surfaces were rinsed twice with 50 mL of sterile deionized water, and the remaining swabbing procedure was as described for the wet surface swabs (Fig. 7).

For the enumeration of *B. cereus* group population in the lettuce leaves and roots, three random plants were selected for sampling, with each plant serving as a replicate. The lettuce was harvested with sterile scissors, and the leaves and roots were placed in separate stomacher bags (Stomacher 400 Classic bags; Seward, London, UK). The samples were mixed with peptone water (Oxoid) in a 1:9 mass ratio and stomached at the maximum speed for 2 min. The *B. cereus group* counts and spore populations on the dry surface swab, leaves, and root samples were enumerated as described above. Fifty milliliter aliquots were taken from the homogenized leaves mixture for downstream microbial composition analysis.

### Investigation of changes to microbial composition in the lettuce leaf microbiome following Bt product treatment

The homogenized leaf mixture was centrifuged at 1,500 × *g* for 10 min to remove large leaf debris. The supernatant was then collected and spun down at 9,000 × *g* for 5 min at 4°C to obtain a pellet. DNA extraction from the pellet was carried out with SPINeasy DNA Kit for Microbiome (MPBio, California, USA) according to the manufacturer's instructions. The quality and quantity of the extracted DNA were assessed with BioDrop DUO (BioDrop). To minimize chloroplast and mitochondrial DNA contamination, peptide nucleic acid (PNA) clamps were added to bind to organelle DNA and block their amplification during PCR (63, 64). Universal mitochondrial PNA clamps (5 μM) (5′ GGCAAGTGTTCTTCGGA 3′) and *Asteraceae* PNA clamps (5′ GGCTCAACTCTGGACAG 3′) were used during the amplification of the 16S rRNA gene V4 region with the forward primer (5′ GTGYCAGCMGCCGCGGTAA 3′) and the reverse primer (5′ GGACTACNVGGGTWTCTAAT 3′). The PCR cycling conditions were as follows: initial denaturation at 95°C for 5 min, 20 cycles at 95°C for 30 s, 75°C for 45 s, 50°C for 1 min, 68°C for 1 min, and a final extension at 72°C for 10 min. Following that, the amplified products were cleaned up with GeneJet PCR Purification Kit (Thermo Fisher Scientific) and sent to Azenta for sequencing of the 16S rRNA gene at the V4 region. The sequences were obtained and then processed in QIIME2, as mentioned previously. The classified ASVs were then used to construct a barplot chart at the genus level using ggplot2 R package (version 3.4.4) (65) in RStudio (version 2023.12.1).

### Statistical analysis

Statistical analyses for bacteria counts were performed using Statistical Package for the Social Sciences for Windows (International Business Machines, New York, USA) when there were three or more datapoints available for each treatment. ANOVA with the Tukey post hoc test was performed to assess significant differences in Caco-2 cell viabilities after B3 or reference strain CFS treatment at each concentration compared to the control. A repeated ANOVA test with a Sidak post hoc test was conducted to assess significant differences in *B. cereus* group counts across 6 weeks within treatments, while an independent (unpaired) *t*‐test was used to determine significant differences in *B. cereus* group counts between treatment and control groups at the same time point. Results were considered statistically significant when *P* < 0.05.

Microbial community statistical analysis was conducted in the QIIME2 environment unless otherwise stated. The α-diversity parameters (Chao1, Shannon index, and Faith’s phylogenetic diversity) were analyzed, and Mann-Whitney *U* test was used to detect statistical differences. Meanwhile, β-diversity of the microbial communities was visualized through canonical analysis of principal coordinates with Bray-Curtis dissimilarity in the vegan R package (version 2.6.8) (66). Potential differences in lettuce leaf microbial composition due to Bt treatment and dispersion were determined through PERMANOVA and PERMDISP, respectively. Differences in community composition and functions were further examined through differential abundance analysis and functional profile analysis via DESeq2 (version 1.42.1) (67) and PICRUSt2 (version 2.5.2) (68) R packages, respectively. The output of PICRUSt2 was visualized with ggpicrust2 (version 1.7.3) (69) R package. Differences with an adjusted *P* value of <0.05 were considered statistically significant.

## Data Availability

The assembled *B. thuringiensis* genome and raw read files of 16S rRNA data were uploaded in NCBI under accession number PRJNA1161229.

## SUPPLEMENTAL MATERIAL

Supplemental material aem.00885-26-s0001.pdf Tables S1 and S2; Fig. S1 and S2.

ASM does not own the copyrights to Supplemental Material that may be linked to, or accessed through, an article. The authors have granted ASM a non-exclusive, world-wide license to publish the Supplemental Material files. Please contact the corresponding author directly for reuse.
