# Supplementary material for: Insights into the food safety implications of a commercial Bacillus thuringiensis product in hydroponic systems
Source: Appl Environ Microbiol. 2026 Jul 10;92(8):e00885-26. doi: 10.1128/aem.00885-26 (PMC13488296; doi:10.1128/aem.00885-26)
Supplement: Supplementary file 1 [file aem.00885-26-s0001.pdf]

Insights into the food safety implications of a commercial *Bacillus thuringiensis* product in hydroponic systems

Supplementary data

Table S1. Genomic composition of assembled genome of *B. thuringiensis* isolate

Feature	Length (bp)	G + C content (%)	Coding sequences	tRNA	rRNA
Chromosome	5 708 468	35.29	6033	107	42
Plasmid pBT-1	418 534	32.68	383	1	0
Plasmid pBT-2	295 555	33.05	295	0	0
Plasmid pBT-3	85 155	34.65	78	0	0
Plasmid pBT-4	84 959	30.72	108	0	0
Plasmid pBT-5	69 396	31.83	86	0	0
Plasmid pBT-6	56 665	34.76	69	0	0
Plasmid pBT-7	12 864	31.51	27	0	0
Plasmid pBT-8	10 657	33.65	14	0	0
Plasmid pBT-9	8 499	29.65	9	0	0
Plasmid pBT-10	5 535	32.81	6	0	0

Table S2. List of *cry* genes annotated within the genome of *B. thuringiensis* isolate by CryProcessor

Cry toxin gene	Gene Location	Start	End	Percent Identity (%)	E value	Bitscore
cry1Ca15	Plasmid pBT-1	104667	108236	100.0	0.0	2437.1
cry1Da1	Plasmid pBT-1	111223	114720	100.0	0.0	2369.7
cry2Ab1	Plasmid pBT-2	24585	26486	100.0	0.0	1253.4
cry1Aa12	Plasmid pBT-2	36135	39527	99.9	0.0	2290.0
cry1Ia10	Plasmid pBT-2	40034	42193	100.0	0.0	1445.3
cry9Ea1	Plasmid pBT-2	49354	52806	100.0	0.0	2323.9
cry1Ac1	Plasmid pBT-3	17469	21005	100.0	0.0	2397.5

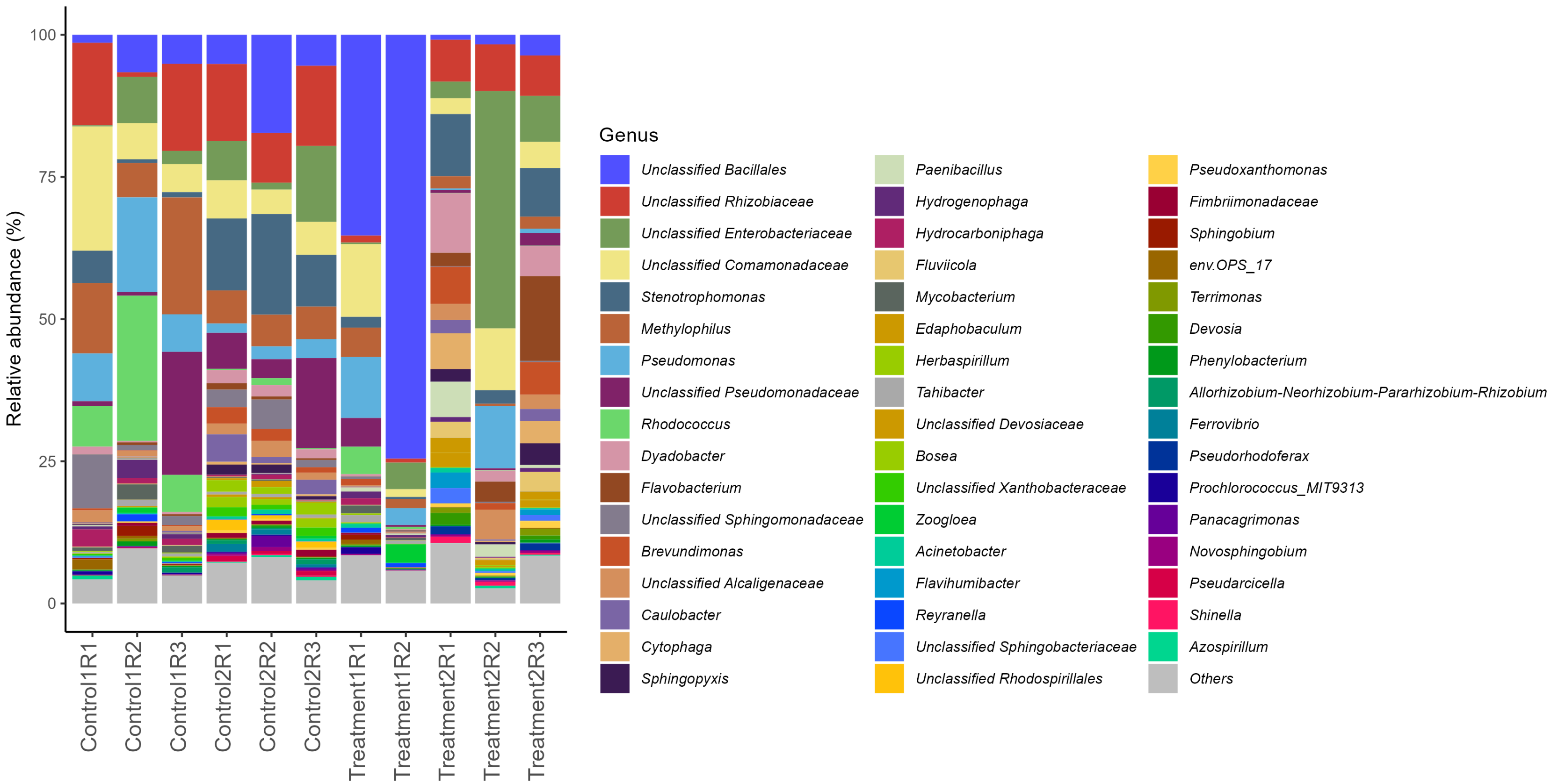

Figure S1. Taxonomic barplot illustrating the relative abundance of the top 50 genera of the microbial community in lettuce leaves at harvest from two independent experiments (n = 6 for each group).

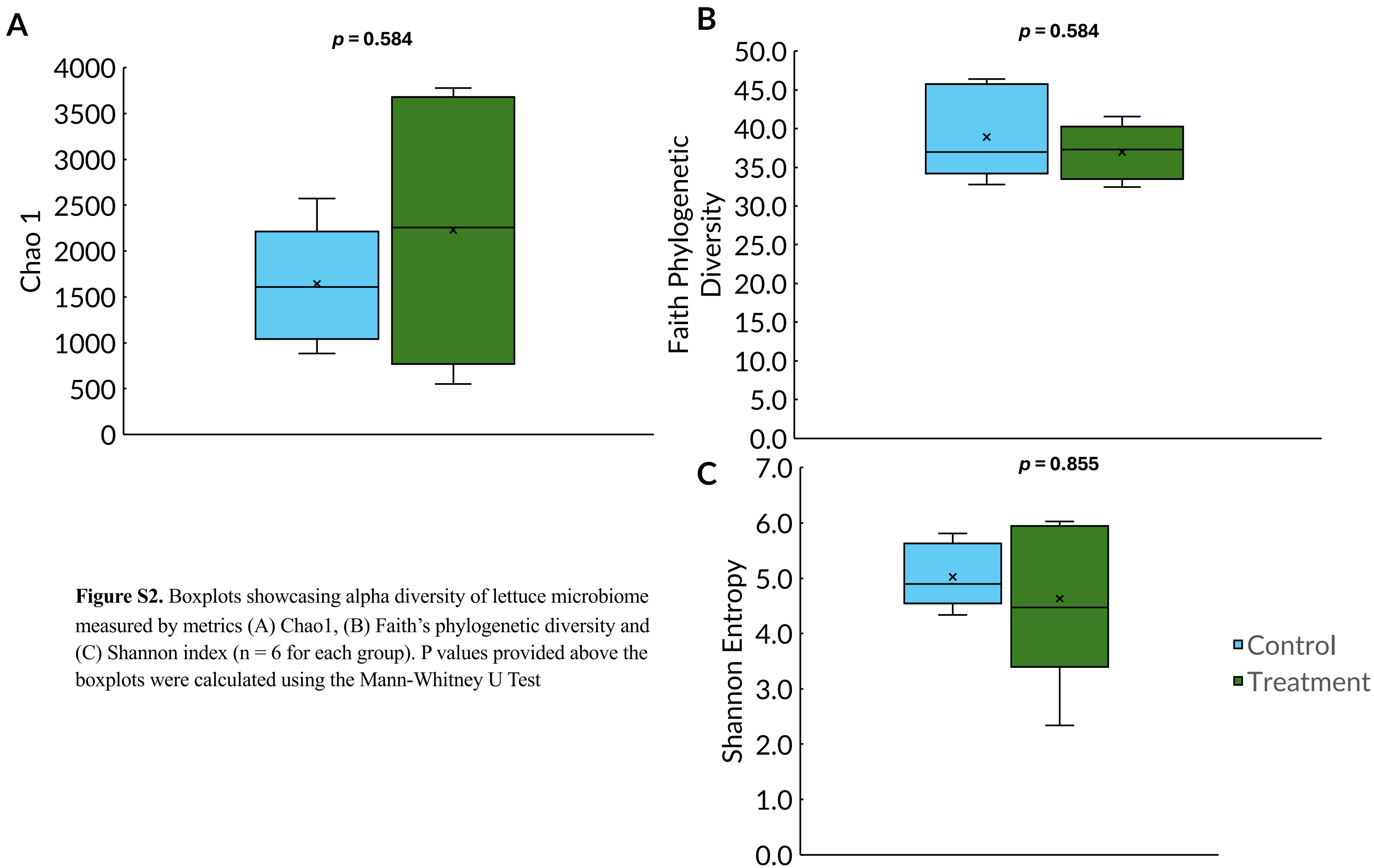

Figure S2. Boxplots showcasing alpha diversity of lettuce microbiome measured by metrics (A) Chao1, (B) Faith's phylogenetic diversity and (C) Shannon index (n = 6 for each group). P values provided above the boxplots were calculated using the Mann-Whitney U Test
